# Therapeutic outcomes of transplantation of amniotic fluid-derived stem cells in experimental ischemic stroke

**DOI:** 10.3389/fncel.2014.00227

**Published:** 2014-08-13

**Authors:** Naoki Tajiri, Sandra Acosta, Gabriel S. Portillo-Gonzales, Daniela Aguirre, Stephanny Reyes, Diego Lozano, Mibel Pabon, Ike Dela Peña, Xunming Ji, Takao Yasuhara, Isao Date, Marianna A. Solomita, Ivana Antonucci, Liborio Stuppia, Yuji Kaneko, Cesar V. Borlongan

**Affiliations:** ^1^Center of Excellence for Aging and Brain Repair, Department of Neurosurgery and Brain Repair, University of South Florida Morsani College of MedicineTampa, FL, USA; ^2^Department of Neurosurgery, Xuanwu Hospital, Capital Medical UniversityBeijing, China; ^3^Department of Neurological Surgery, Okayama University Graduate School of Medicine, Dentistry and Pharmaceutical SciencesOkayama, Japan; ^4^Laboratory of Molecular Genetics, DISPUTer, School of Medicine and Health Sciences, “G. d ’Annunzio” UniversityChieti-Pescara, Italy

**Keywords:** cerebral ischemia, neural stem/progenitor cells, regenerative medicine, neurogenesis, neurotrophic factors

## Abstract

Accumulating preclinical evidence suggests the use of amnion as a source of stem cells for investigations of basic science concepts related to developmental cell biology, but also for stem cells’ therapeutic applications in treating human disorders. We previously reported isolation of viable rat amniotic fluid-derived stem (AFS) cells. Subsequently, we recently reported the therapeutic benefits of intravenous transplantation of AFS cells in a rodent model of ischemic stroke. Parallel lines of investigations have provided safety and efficacy of stem cell therapy for treating stroke and other neurological disorders. This review article highlights the need for investigations of mechanisms underlying AFS cells’ therapeutic benefits and discusses lab-to-clinic translational gating items in an effort to optimize the clinical application of the cell transplantation for stroke.

## Why is there a need for novel treatments in stroke?

Stroke, the fourthleading cause of death and the leading cause of disability in the United States (Roger et al., 2012), has only one FDA-approved drug, namely tissue plasminogen activator (tPA). Due to tPA limitations and complications, which include a limited therapeutic window (4.5 h from disease onset to tPA administration) and adverse effects associated with delayed treatment (i.e., hemorrhagic transformation), only a mere 3 percent of ischemic stroke patients actually benefit from the tPA treatment (Graham, 2003; Yip and Demaerschalk, 2007). This significant unmet clinical need for stroke has prompted investigations to increase the therapeutic window with innovative treatment strategies specifically targeting the restorative phase, which begins days to weeks post-stroke (Matsukawa et al., 2009; Yu et al., 2009; Antonucci et al., 2011; Kaneko et al., 2011; Manuelpillai et al., 2011).

## Do stem cells exist in the amnion?

Stem cells have emerged as a prospective restorative agent for stroke due to their ability to abrogate sub-acute and chronic secondary cell death associated with the disease (Borlongan et al., 1997; Nishino and Borlongan, 2000). We previously reported the isolation of viable rat amniotic fluid-derived stem (AFS) cells (Tajiri et al., 2012). Together with other research teams, the grafted stem cells’ production of trophic factors and cytokines, as well as the increase in levels of neurotrophic factors and reduced inflammatory response in the ischemic stroke region, have been directly attributed to the positive effects by transplantation of AFS cells (Yu et al., 2009; Antonucci et al., 2011; Kaneko et al., 2011; Manuelpillai et al., 2011). Furthermore, the inhibition of apoptosis and oxidative stress, in tandem with stimulation of angiogenesis, neurogenesis, and synaptogenesis, may be linked as a benefit of AFS cells against stroke deficits (Yu et al., 2009; Antonucci et al., 2011; Kaneko et al., 2011; Manuelpillai et al., 2011). Even though stem cells can be harvested from various sources (Borlongan et al., 2004, 2005; Hematti et al., 2004; Clavel and Verfaillie, 2008; Burns et al., 2009; Ou et al., 2010), including bone marrow, fetal and embryonic tissues, amnion-derived stem cells are an appealing choice because of many logistical and ethical advantages including the ease of isolation of the stem cells from amnion tissue and the fluid (Yu et al., 2009; Antonucci et al., 2011; Kaneko et al., 2011; Manuelpillai et al., 2011). Similar to amniotic-tissue derived cells, the harvest of AFS cells poses negligible risk of injury to the fetus. These cells are isolated from amniotic fluid collected from amniocentesis, a pre-natal exam performed at around 15–20 weeks of gestation. Accordingly, since AFS cells can be isolated much earlier, compared to amniotic tissue-derived cells, AFS cells possess properties that closely resemble embryonic and mesenchymal cell markers, which could be more beneficial at treating diseases. The low immunogenicity, low tumorgenicity, high proliferative capacity and anti-inflammatory characteristics, are phenotypic features of transplantable cells (Newman et al., 2005) that support AFS cells to be a safe and effective donor cell for stroke (Yu et al., 2009; Antonucci et al., 2011; Kaneko et al., 2011; Manuelpillai et al., 2011). Another distinguishing feature of AFS cells when compared to amniotic tissue-derived cells is the sterility involved. AFS cells are harvested via amniocentesis under aseptic condition, but the sterility may be compromised when stem cells are extracted from amnion tissue during child delivery.

AFS cells can phenotypically commit to various lineages (Prusa and Hengstschlager, 2002; In’t Anker et al., 2003; Prusa et al., 2003; Fauza, 2004; Tsai et al., 2004, 2006; McLaughlin et al., 2006). A misleading concept is the term “fluid” associated with AFS cells because cells isolated during amniocentesis are comprised of multiple stem cells originating from extra-embryonic and embryonic tissues (Prusa and Hengstschlager, 2002) and their properties differ with gestational age (Yu et al., 2009; Antonucci et al., 2011; Kaneko et al., 2011; Manuelpillai et al., 2011). Accordingly, phenotypic characterization of AFS cells reveal a plethora of stem cell subtypes, from pluripotent embryonic stem cells to multipotent adult stem cells owing likely to the age-dependent tissue plasticity potential (De Coppi et al., 2007; Mauro et al., 2010). Indeed, AFS cells from second trimester amniotic fluid display the ability to differentiate into all three germ layers and express Oct-4, Nanog, and SSEA-4 (Roubelakis et al., 2007), which are pluripotent embryonic stem cell markers (Yu et al., 2009; Antonucci et al., 2011; Kaneko et al., 2011; Manuelpillai et al., 2011). Although considered as “adult stem cells”, the doubling time for the AFS cells population is approximately 30–36 h with a high regeneration capacity that can be extended for over 250 doublings without any measurable loss of chromosomal telomere length (De Coppi et al., 2007). Altogether, these studies support the amnion as a potent source of stem cells for investigations of basic science concepts related to developmental cell biology, but also for therapeutic purposes such as AFS cell transplantation for treating human disorders, especially stroke.

## Are AFS cells transplantable and do they exert functional benefits?

The following protocol allows the isolation of AFS cells from isolated amniotic fluid samples obtained from timed pregnant Sprague-Dawley rats at gestation age 16–18 weeks (Tajiri et al., 2012). For each sample, 2–3 ml of amniotic fluid, corresponding to a cell number ranging from 2 × 10^3^ to 2 × 10^6^ are centrifuged for 10 min at 1800 rpm. The high variance in the amount of cells which can be isolated from amniotic fluid is influenced by factors such as child birth, genetics, and fluid cell isolation. It is expected, however, that these discrepancies can be overcome when technology is able to be standardized with appropriate quality control and assurance. This will yield a more accurate and efficient cell isolation in the near future. Pellets are then resuspended in Iscove’s modified Dulbecco’s medium supplemented with 20% FBS, 100 U/ml penicillin, 100 μg/ml streptomycin (Sigma), 2 mM L-glutamine, 5 ng/ml basic fibroblast growth factor (FGF2) and incubated at 37°C with 5% humidified CO_2_. After 7 days, non-adherent cells are removed and the adherent cells are allowed to grow in the same medium, which is changed every 4 days. When the cell culture reaches confluency (about 20 days after the primary culture), cells are treated with 0.05% trypsin and 0.02% EDTA, then counted and replaced in 25 cm^2^ culture flasks. For routine AFS cell procedure, an approximate yield of about 22 million stem cells per amnion fluid aspirate is anticipated. The viability yield of AFS cells is about 70%, which would be an estimated 15 million stem cells per amnion fluid aspirate. An estimated range of several million AFS cells per milliliter of amnion fluid are therefore expected to be obtained.

This protocol allows ample AFS cells for transplantation studies. Indeed, accumulating preclinical data have demonstrated the potential of transplantation of AFS cells for treating experimental models of brain diseases (Yu et al., 2009; Antonucci et al., 2011; Kaneko et al., 2011; Manuelpillai et al., 2011). For example, AFS cells are a potentially valuable source of stem cells to treat Parkinson’s disease because under standard neuronal induction protocols for stem cells, AFS cells preferentially differentiate into a dopaminergic phenotype (Pisani et al., 2005). In parallel, transplantation of AFS cells has been examined in stroke, with AFS transplanted ischemic stroke mice exhibiting reduced short-term memory impairment and improved sensorimotor ability, somatosensory functions, and motor coordination (Rehni et al., 2007). Although this study shows the beneficial effects of AFS cell transplantation in experimental stroke, the mechanism underlying the observed therapeutic benefits remained underexplored. Perhaps AFS cells are mediating specific neurotransmitter release to restore cellular function in an injured brain (Broughton et al., 2013). This may explain a reversal of disease-induced impairment of memory following AFS cell transplantation in an ischemic model of stroke when transfer latency time (TLT) is used as a parameter of memory. If administration of AFS cells following middle cerebral artery occlusion and reperfusion significantly attenuated ischemia-reperfusion induced increase in day 7 TLT, it is likely that the AFS cells are secreting the neurotransmitters necessary to ameliorate memory and subsequently, cognition in general (Rehni et al., 2007).

## How do AFS cells afford repair of the stroke brain?

Our group transplanted AFS cells in experimental stroke animals (via occlusion of the middle cerebral artery; Borlongan et al., 1998) using motor and cognitive tests, and subsequent histological analysis of the brain to assess the mechanism of action associated with AFS cell therapy for stroke. Although still impaired compared to sham-operated animals (Acosta et al., 2010), intravenous transplantation of AFS cells (1 million viable cells) in adult Sprague-Dawley rats at 30 days after experimental stroke revealed that motor and cognitive deficits were reduced at 60 days post-MCAo compared to vehicle-infused stroke animals (Tajiri et al., 2012). Moreover, histological analyses revealed that the AFS cell-transplanted stroke animals significantly decreased infarct volumes by 92% compared to the vehicle-infused stroke animals, which likely facilitated the recovery of motor and cognitive functions. In addition, cell proliferation was significantly upregulated in the neurogenic subventrical zone of the AFS cell-transplanted stroke animals compared to the vehicle-infused stroke animals. In tandem, immature neuronal cells also increased in the subventrical zone of AFS cell-transplanted stroke animals compared to the vehicle-infused stroke animals. The increased cell proliferation and reduced neuronal loss in the subventrical zone was similarly observed in the dentate gyrus, another major neurogenic niche. That AFS cell transplantation attenuated stroke-induced behavioral and histological deficits coincided with increased cell proliferation and neuronal differentiation in the two neurogenic sites, namely subventricular zone and dentate gyrus, implicates a major role of graft-induced host tissue repair in the brain remodeling process following stroke (Table 1).

**Table 1:** **Effects of AFS cells and amniotic tissue-derived cell in stroke-related disease**.

**Protective effects of AFS and amniotic tissue-derived stem cells in stroke-related disease**	**Reference**
Stroke rats manifest a robust reduction of infarct volumes by 92% and reduced local inflammation.	Liu et al. (2008), Tao et al. (2012) and Broughton et al. (2013).
Cell proliferation, neuronal differentiation, and immature neuronal cells significantly upregulated in the subventrical zone and dentate gyrus of stroke rats.	Ekdahl et al. (2009), Jezierski et al. (2010), Zhang et al. (2010) and Prasongchean et al. (2012).
Reduced short term memory impairment and improved sensorimotor ability, somatosensory functions, and motor coordination in stroke rats.	Rehni et al. (2007) and Broughton et al. (2013).
Ischemic rats present an increase of neurogenesis in the hippocampus, leading to improved reference memory.	Tajiri et al. (2012).
Reversal of hemi-parkisonian syndrome and behavioral improvement thanks to the formation of new dopaminergic fibers in the denervated striatum.	Sheng et al. (1993) and Bankiewicz et al. (1994).
Rats subjected to middle cerebral artery occlusion model express MAP2, Nestin, and glial fibrillary acidic protein that improve behavioral recovery.	Liu et al. (2008) and Tao et al. (2012).
The administration of melatonin significantly increases the proliferation and survival of human amniotic epithelial cells and boosts neuronal differentiation.	Kaneko et al. (2011).
Human amniotic membrane-derived mesenchymal stem cells lack major histocompatibility complex class I molecule greatly reducing transplant rejection.	Tao et al. (2012) and Broughton et al. (2013).
Amniotic membrane mesenchymal stem cells have innate capacity to express factors for endothelialization and angiogenesis. Crucial for wound recovery in ischemic diseases.	Warrier et al. (2012) and Broughton et al. (2013).
Amniotic membrane cell grafts enhance the recovery of cardiac function.	Cargnoni et al. (2009).

*A synopsis of the beneficial behavioral and cognitive effects observed in experimental animals models of stroke*.

## Can we translate AFS cell graft-mediated functional recovery in experimental stroke to the clinic?

Many stroke victims present with symptoms characterized by sensorimotor and cognitive functions (Grefkes et al., 2008; Rush et al., 2010; Lin et al., 2011). Up to now, most stroke animal models have focused on motor impairments, disregarding the cognitive declines that proceed after the brain offense (Borlongan, 2009; Chopp et al., 2009; STEPS, 2009). Findings on the pathology of ischemic stroke support that secondary cell death may extend beyond the routine cortical damage towards the hippocampus, which is the key brain structure for learning and memory consolidation (Scoville and Milner, 1957; Shors et al., 2002; Saxe et al., 2006; Dupret et al., 2008). A decline in adult neurogenesis is magnified during aging and correlates with worsening of cognitive functions (McDonald and Wojtowicz, 2005; Drapeau and Nora Abrous, 2008; Encinas et al., 2011). Interestingly, the impaired neurogenesis and the cognitive decline in the aging hippocampus (Freret et al., 2009; Dhawan et al., 2010; Zvejniece et al., 2012) also accompany stroke (Dhawan et al., 2010; Wattanathorn et al., 2011; Zvejniece et al., 2012).

Whereas our cognitive test results did not show any difference in learning performance between the transplanted and vehicle-infused stroke animals, the AFS cell-transplanted stroke animals demonstrated a significantly improved reference memory compared to the vehicle-infused stroke animals. This improvement in the memory task directly correlates with increased neurogenesis in the hippocampus of AFS cell-transplanted stroke animals relative to vehicle-infused stroke animals. In order to increase the clinical relevance of cell therapy for stroke, especially when evaluating cognitive deficits, is to employ a hippocampal dependent task for spatial memory and reference memory (Gallagher et al., 1993; Duva et al., 1997; Clarke et al., 1999; Anisman and McIntyre, 2002; Broadbent et al., 2004; Clark et al., 2007; Gillani et al., 2010; Bergado et al., 2011; Jurgens et al., 2012), and long-term potentiation (Bliss and Collingridge, 1993; Norris and Foster, 1999; Gusev and Alkon, 2001; Richardson et al., 2002; Vorhees and Williams, 2006; Pisu et al., 2011; Xu et al., 2012). Our data advanced the notion that transplanted AFS cells selectively aid in the recovery of the reference memory, but not task acquisition. Cognitive improvements in stem/progenitor cell transplanted stroke animals have been linked previously with the decline in cerebral infarct volumes (Nishino et al., 1993; Fukunaga et al., 1999, 2003; Mimura et al., 2005), as well as reduced secondary cell death loss in brain areas known to modulate motor and cognitive functions (Ebrahimi et al., 1992; Pisani et al., 2005; Takahashi et al., 2008; Tabuse et al., 2010; Miyoshi et al., 2012). That the AFS cell transplanted stroke animals displayed improved motor and cognitive performance, reduced the brain infarcts and the secondary cell death, and enhanced the level of neurogenesis provide insights on possible mechanisms of action underlying the functional benefits afforded by AFS cell transplantation. Our observed transplant-mediated recovery of motor and cognitive functions confirms a recent report of AFS therapeutic effects in stroke (Rehni et al., 2007), but our study expanded the short timeline of 7 days from the previous study to 63 days.

The transplantation of AFS cells shows extensive therapeutic benefits such as reduction in infarct volume, enhanced cell proliferation, increased neuronal differentiation, and improved memory and motor skills, seen with other stem cells (Yasuhara et al., 2006a,b, 2008; Hara et al., 2007). The exact underlying mechanism of these benefits is still unknown and understanding therapeutic pathways could potentially yield promising insights into optimizing AFS cell transplantation towards the clinical trial stage. Clinical trials that closely reflect the preclinical data on safety and efficacy of AFS cells in animal models of stroke will enhance the functional outcome of this cell therapy in stroke patients. Based on preclinical data demonstrating AFS cell safety and efficacy, patients who suffer a stroke and show significant inflammation of the brain or who display short-term memory loss due to the accompanying injury to the hippocampus seem to be the best candidates for future clinical trials of AFS cells. Moreover, AFS cell therapy trials could be extended to patients who have suffered from cardiac stroke, as animal models show promising improvement in cardiac functions following AFS cell transplantation (Bollini et al., 2011). In addition, a better understanding of the mechanism(s) involved in the functional improvement seen in transplanted stroke animals will likely further optimize AFS cell therapy.

When contemplating with clinical applications of AFS cells, the route of transplantation is key to successful outcomes of targeted stroke patient population. Intracerebral (Rehni et al., 2007; Liu et al., 2008), intraperitoneal (Ghionzoli et al., 2010), and intravenous (Tajiri et al., 2012) have been explored in AFS transplantation in experimental models of cerebral ischemia. The intravenous route of transplantation is a minimally invasive procedure and poses less risk to the patient compared to intracerebral transplantation. A peripheral injection method may be favored over the direct transplantation route so that the stem cells can be administered quickly after the onset of a stroke. A limited FDA-approved clinical trial for intravenous transplantation of placenta/amnion-derived stem cells in sub-acute stroke patients was terminated with no available safety and efficacy results (ClinicalTrials.gov., 2014).

The recommendations of Stem cell Therapeutics as an Emerging Paradigm for Stroke (STEPS; Borlongan, 2009; Chopp et al., 2009; STEPS, 2009) are likely to facilitate the translational research on AFS cell grafts for stroke. Such translational blueprint for cell therapy in stroke provides important guidance in experimental stroke testing and stem cell therapy, including the need for multiple strains, both genders, and age groups to evaluate safety and efficacy of AFS cells (Borlongan, 2009; Chopp et al., 2009; STEPS, 2009). Equally important is the phenotypic characterization of AFS cells in order to allow replications of the results, but also to maintain quality control and quality assurance of the cells for transplantation therapy in the clinic. With this in mind, another key component of translating laboratory studies into clinical applications is to carefully and critically analyze the type of transplantation procedure to be employed in the clinic. The efficient isolation of AFS cells (i.e., during amniocentesis) (Kalogiannidis et al., 2011) may prove applicable to allogenic grafts in the stroke patients. The animal model shows the efficacy of AFS cells 30 days after stroke, which suggests its beneficial application to chronic stroke patients.

The collection of the AFS cells will likely require banking of cells associated with storage costs that may not be covered by health insurance. This will in turn require the donor to shoulder the costs ranging to thousands of dollars. The studies conducted so far exploring the immunogenic properties of human amniotic cells have revealed that these cells inhibit the function of different immune cells of the innate and adaptive immune response (Borlongan et al., 1996; Insausti et al., 2014); however, it is also documented that even autologous grafts have the potential to elicit an immune response. Therefore, further investigations to determine whether AFS cells are truly immune-free need to be conducted. In addition, the heterogenic population of AFS cells contains many cell phenotypes. While such variety of cell lineages suggests differentiation capability of AFS cells to multiple cell phenotypes and thus allowing many disease indications for AFS cell therapy, the stability of phenotypic expression and functional effects of AFS cells remain to be fully investigated. Altogether, these challenges may seem to hinder translational potential of AFS cells, but they pose as open avenues of research waiting to be discovered towards realizing the full potential of AFS cells for transplant therapy.

It is also important to discuss the practical issues associated with stroke therapy in adults. The unpredictable occurrence of a stroke event can be addressed by the ready availability of AFS cells. However, the route of stem cell administration may be dictated by the stroke disease phase. For example, acute stroke will require rapid delivery of cells via minimally invasive route, while chronic stroke may be amenable to intracerebral delivery of the cells. In addition, the ready availability of the amnion cells and timing of transplantation for both acute and chronic phases of stroke will require cryopreserved cells, thereby requiring high viability of cells following freezing and thawing storage. In this case, a panel of cell release criteria monitoring high viability of cells, normal karyotyping, and phenotypic stability (among other cell quality assurance issues) need to be in place prior to transplantation of AFS cells as these are common cell release criteria in place for other donor stem cell types. Ultimately, the maintenance of therapeutic efficacy due to scarce data to date providing “efficacy” readout for cells prior to transplantation is a challenge. A simple assay of neurotransmitter release or growth factor secretion after thawing may need to be developed as an efficacy release criterion which should enhance the therapeutic outcome of AFS cell transplantation.

Transplanted AFS cells-induced cell proliferation, in tandem with a decrease in neuronal loss, produced robust reduction in cerebral infarcts (Tajiri et al., 2012). The subventricular zone and dentate gyrus, two neurogenic niches in the brain, are critical to the repair of damaged brain tissue (Ekdahl et al., 2009; Jezierski et al., 2010; Zhang et al., 2010; Prasongchean et al., 2012). The results imply that AFS cell transplantation may have enhanced endogenous repair mechanisms by maximizing the potential of these two neurogenic sites to confer a host brain remodeling process. Intravenous AFS cell transplantation in the chronic stage of experimental stroke decreased motor and cognitive impairments, maintained cell proliferation, and increased cell differentiation in the host brain (Tajiri et al., 2012). The mechanism of action appears to involve AFS grafted cells’ capacity to solicit endogenous stem cells for brain repair. These observations offer insights into therapeutic pathways of brain repair, and guide the design of clinical trials of cell therapy in stroke.

## Conflict of interest statement

CVB holds patents and has pending patents in stem cell biology and applications.
